# Prognostic value of an autophagy-related gene expression signature for endometrial cancer patients

**DOI:** 10.1186/s12935-020-01413-6

**Published:** 2020-07-13

**Authors:** Hui Wang, Xiaoling Ma, Jinhui Liu, Yicong Wan, Yi Jiang, Yankai Xia, Wenjun Cheng

**Affiliations:** 1 Department of Gynecology, The First Affiliated Hospital of Nanjing Medical University, 368 North Jiangdong Road, Nanjing, 210029 Jiangsu People’s Republic of China; 2 State Key Laboratory of Reproductive Medicine, Center for Global Health, School of Public Health, Nanjing Medical University, Nanjing, 211166 China; 3 State Key Laboratory of Modern Toxicology of Ministry of Education, School of Public Health, Nanjing Medical University, 101 Longmian Avenue, Nanjing, Jiangsu 211166 China

**Keywords:** Autophagy-related genes, Endometrial cancer, Prognosis

## Abstract

**Background:**

Autophagy is associated with cancer development. Autophagy-related genes play significant roles in endometrial cancer (EC), a major gynecological malignancy worldwide, but little was known about their value as prognostic markers. Here we evaluated the value of a prognostic signature based on autophagy-related genes for EC.

**Methods:**

First, various autophagy-related genes were obtained via the Human Autophagy Database and their expression profiles were downloaded from The Cancer Genome Atlas. Second, key prognostic autophagy-related genes were identified via univariate, LASSO and multivariate Cox regression analyses. Finally, a risk score to predict the prognosis of EC was calculated and validated by using the test and the entire data sets. Besides, the key genes mRNA expression were validated using quantitative real-time PCR in clinical tissue samples.

**Results:**

A total of 40 differentially expressed autophagy-related genes in EC were screened and five of them were prognosis-related (CDKN1B, DLC1, EIF4EBP1, ERBB2 and GRID1). A prognostic signature was constructed based on these five genes using the train set, which stratified EC patients into high-risk and low-risk groups (p < 0.05). In terms of overall survival, the analyses of the test set and the entire set yielded consistent results (test set: p < 0.05; entire set: p < 0.05). Time-dependent ROC analysis suggested that the risk score predicted EC prognosis accurately and independently (0.674 at 1 year, 0.712 at 3 years and 0.659 at 5 years). A nomogram with clinical utility was built. Patients in the high-risk group displayed distinct mutation signatures compared with those in the low-risk group. For clinical sample validation, we found that EIF4EBP1and ERBB2 had higher level in EC than that in normal tissues while CDKN1B, DLC1 and GRID1 had lower level, which was consistent with the results predicted.

**Conclusions:**

Based on five autophagy-related genes (CDKN1B, DLC1, EIF4EBP1, ERBB2 and GRID1), our model can independently predict the OS of EC patients by combining molecular signature and clinical characteristics.

## Background

As an evolutionarily ancient and highly conserved biological behavior, autophagy plays a cytoprotective role in eukaryotic cells through degrading unnecessary or dysfunctional organelles under the condition of hypoxia, starvation, nutrition deficiency or high PH [1]. However, excessive self-degradation can be poisonous [2]. Accordingly, autophagic dysfunction involved in numerous human pathologies, such as liver and heart disease, neurodegeneration, muscle disease, and cancer [3]. However, the mechanisms of autophagy in cancer remain stagnantly understood. A clearer eyesight into these mechanisms may facilitate the invention of autophagy-focused therapeutic interventions.

Endometrial cancer (EC) is one of the most common malignant diseases in women worldwide, with an estimated 61,880 newly-diagnosed cases and 12,160 deaths in the United States in 2019 [4]. Primarily treated with surgical resection, the prognosis of women with early-stage EC is excellent, with a 5-year survival rate of over 70% [5]. However, 15–20% of cases still show post-operative vaginal or pelvic EC relapse, often metastasizing to distant sites [6]. Despite the advance in surgical techniques, chemotherapy, or radiotherapy, data show that the EC mortality of EC has bypassed EC incidence [7]. To further improve the outcomes of EC treatments, physicians need to identify high-risk patients and tailor precise treatment.

Recent studies revealed associations between autophagy and pathophysiological processes in EC. The so-called “stone-like” structure (SLS) has emerged as the hallmark of autophagic activity. SLSs are detected exclusively in EC and rarely in atypical hyperplasias, suggesting that autophagy is more active in EC cells than in normal or hyperplastic endometrial cells [8]. Targeting autophagy enhances sorafenib cytotoxicity and inhibits tumor growth and pulmonary metastasis, which can help us gain insights into the unopposed resistance of advanced EC to sorafenib and provide a possibility to develop a new therapeutic intervention for recurrent EC [9]. However, little literature is available regarding the prediction of EC prognosis based on its molecular mechanisms or biological behavior.

In the present study, we examined the relevance between autophagy-related genes (ARGs) expression profiles and clinical outcomes in 552 EC patients from The Cancer Genome Atlas (TCGA). Then, using ARGs as an independent risk factor, a prognosis-prediction model was established for EC overall survival (OS). Results from this study could provide an autophagy-targeted strategy for predicting and monitoring the prognosis of patients with EC.

## Methods

### Collection of data

The Human Autophagy Database (http://www.autophagy.lu/index.html) is a public repository containing information about the human genes involved in autophagy. Various ARGs were obtained via this database. RNA sequencing (RNA-seq) data of ARGs from each individual and their clinical information were downloaded from The Cancer Genome Atlas (TCGA) data portal (http://cancergenome.nih.gov/).

### Identification of differentially expressed ARGs

The limma package in R software was used to search for differentially expressed ARGs between EC and non-tumor samples. Genes exhibiting at least onefold change and an FDR < 0.05 were regarded as obviously differentially expressed. Then, their most involved biological functions and pathways were revealed by a series of gene functional enrichment analyses, including Gene ontology (GO) and Kyoto Encyclopedia of Genes and Genomes (KEGG) at Enrichr (http://amp.pharm.mssm.edu/Enrichr/) [10].

### Calculation of a risk score based on ARG model

ARGs expression profiles were downloaded from TCGA and normalized by [log2(data + 1)]. The entire set was randomly separated into the train set and the test set in a ratio of 6 to 4. Using the gene expression profile of the train set, ARGs were submitted to univariate, LASSO Cox regression and multivariate analyses, to remove the genes which might not be an independent indicator in EC prognosis. Time-dependent receiver operating characteristic (ROC) analysis was used to investigate the prognosis-prediction accuracy and risk score of each ARG. The area under the curve (AUC) with different cutoffs was used to measure prognosis-prediction accuracy. Finally, several prognostic ARGs were screened out, and the coefficient of each prognostic gene in the risk model was assessed with corresponding multivariate analysis. EC patients were then divided into high- and low-risk groups by the cutoff medians, which was calculated according to the expression level of ARGs and the estimated regression coefficient. To investigate whether the risk score could be used as an independent predictor of OS in the TCGA cohort of EC patients, the univariate and multivariate Cox regression analyses were conducted. Clinicopathologic characteristics of EC patients were downloaded from TCGA database (Table 1). The risk score, age, clinical stage, grade, peritoneal wash, radiation therapy, surgical approach, cancer status, and histological type were used as covariates. The test set and the entire set were used to validate this signature and a nomogram was constructed. This nomogram was corrected by calibration curves.

**Table 1:** Clinicopathologic characteristics of 342 EC patients from TCGA database

Characteristics	Entire set (n = 342)
N (%)	
Age, years	
≤ 60	141 (41.2%)
> 60	201 (58.8%)
Stage	
I	221 (64.6%)
II	31 (9.1%)
III	77 (22.5%)
IV	13 (3.8%)
Grade	
G1	80 (23.4%)
G2	74 (21.6%)
G3	188 (55.0%)
Peritoneal wash	
Negative	293 (85.7%)
Positive	49 (14.3%)
Person neoplasm cancer status	
Tumor free	291 (85.1%)
With tumor	51 (14.9%)
Radiation therapy	
Yes	140 (40.9%)
No	202 (59.1%)
Histological type	
Endometrioid endometrial adenocarcinoma	268 (78.4%)
Serous endometrial adenocarcinoma	61 (17.8%)
Mixed serous and endometrioid	13 (3.8%)
Surgical approach	
Minimally invasive	150 (43.9%)
Open	192 (56.1%)

### Gene Set Enrichment Analysis

Gene Set Enrichment Analysis (GSEA) was set up in TCGA series using a molecular signature database (MSigDB) c2.cp.kegg.v6.2.symbols.gmt gene sets [11]. The GSEA, visualized in Enrichment Map software and Cytoscape [12], was applied to determine whether the members of a given gene set were significantly correlated with our risk score.

### Somatic mutation analysis

Package “maftools” was used to summarize, analyze, annotate and visualize mutation annotation format (MAF) files [13]. The T test was applied to identify the differentially mutated genes between high-risk and low-risk groups. The plotmaf Summary function was employed to plot the numbers and types of various MAF files. OncoPlot was used to plot the top ten most obviously mutated genes.

#### Preparation for human tissue samples

This research was approved by the Institutional Review Board of Nanjing Medical University and informed consent was obtained from all subjects. Tissue samples were collected from patients after surgery at the First Affiliated Hospital of Nanjing Medical University from 2017 to 2019 and then stored at − 80 °C, including 15 EC tissues and 15 normal endometrial tissues.

#### RNA isolation and quantitative real-time PCR (qRT-PCR)

Total RNA from human tissues were extracted separately using Trizol reagent (Invitrogen, USA). The integrity of isolated RNA was assessed by NanoDrop 2000 Spectrophotometer (Thermo Scientific, Wilmington, DE, USA). RNA was reversely transcribed into cDNA using the high capacity reverse transcription kits (TaKaRa, Shiga, Japan) and qRT-PCR was performed in the LightCycler480II (Roche) using SYBR Green technology (Takara). The relative expression level was calculated by comparing the 2-ΔΔCt method. The primers for GAPDH, CDKN1B, DLC1, EIF4EBP1, ERBB2 and GRID1 were purchased from Tsingke (NanJing, China). The forward primer of GAPDH is CGCTCTCTGCTCCTCCTGTT. The reverse primer of GAPDH is CATGGGTGGAATCATATTGG. The forward primer of CDKN1B is AACGTGCGAGTGTCTAACGG. The reverse primer of CDKN1B is CCCTCTAGGGGTTTGTGATTCT. The forward primer of DLC1 is CCACGGACCTCCCATCTTC. The reverse primer of DLC1 is GCTGTGCATACTGGGGGAA. The forward primer of EIF4EBP1 is CTATGACCGGAAATTCCTGATGG. The reverse primer of EIF4EBP is CCCGCTTATCTTCTGGGCTA. The forward primer of ERBB2 is TGCAGGGAAACCTGGAACTC. The reverse primer of ERBB2 is ACAGGGGTGGTATTGTTCAGC. The forward primer of GRID1 is TGGCTGTGCATCTGCCAAT. The reverse primer of GRID1 is CGTAGAACATGACGAACTTCTGC. All procedures for qRT-PCR were performed according to the manufacturer’s protocol and all experiments were repeated three times.

### Statistical analysis

Univariate and multivariate Cox regression analyses were used to select the survival-related ARGs and construct the risk score model. The survival curves were plotted by Kaplan–Meier (K–M) method and differences in the survival rates between high- and low-risk groups were evaluated by the log-rank test. ROC curve and AUC were plotted by SurvivalROC package in R. p-value less than 0.05 was considered statistically significant.

## Results

### Identification of differently expressed ARGs

**Fig. 1:**
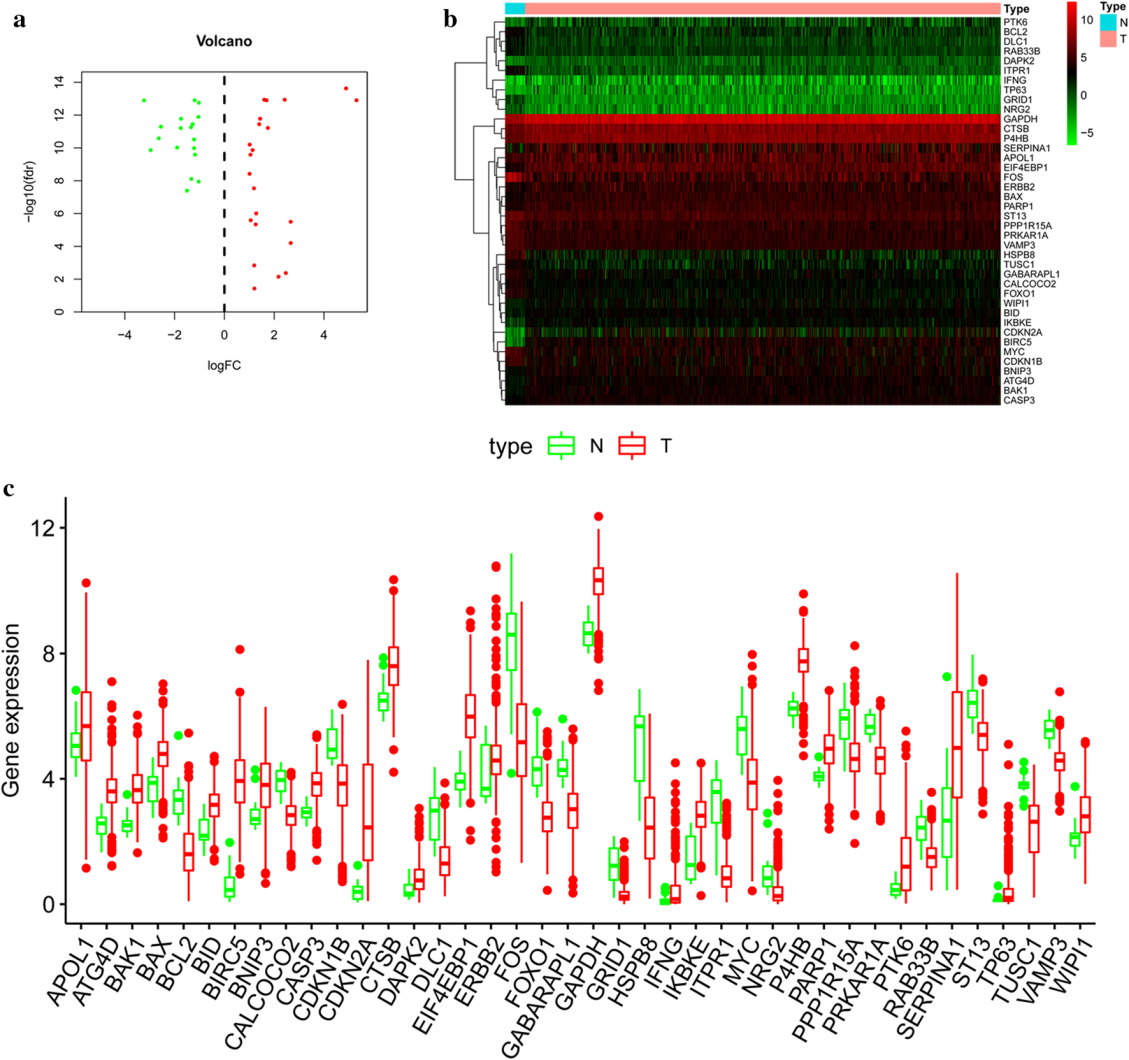
Differentially expressed autophagy-related genes (ARGs). Volcano plot (**a**) and heatmap (**b**) demonstrating differentially expressed genes between endometrial cancer and normal endometrial tissues. Red shows high expression and green low expression. **c** The expression patterns of 40 ARGs in EC types and paired non-tumor samples. Each red dot represents a distinct tumor sample and green a non-tumor sample

RNA-seq and clinical data of 552 EC tissue samples and 23 non-tumor samples were downloaded from TCGA. With |log2(Fold Change)| > 1 and FDR < 0.05, we finally obtained 22 up-regulated and 18 down-regulated ARGs between EC and non-tumor tissues (Fig. 1a, b). Box plots were visualized to display the expression patterns of the 40 differentially expressed ARGs, including 22 up-regulated genes (APOL1, ATG4D, BAK1, BAX, BID, BIRC5, BNIP3, CASP3, CDKN2A, CTSB, DAPK2, EIF4EBP1, ERBB2, GAPDH, IFNG, IKBKE, P4HB, PARP1, PTK6, SERPINA1, TP63, WIPI1) and 18 down-regulated genes (BCL2, CALCOCO2, CDKN1B, DLC1, FOS, FOXO1, GABARAPL1, GRID1, HSPB8, ITPR1, MYC, NRG2, PPP1R15A, PRKAR1A, RAB33B, ST13, TUSC1, VAMP3) (Fig. 1c).

### Functional annotation of the differentially expressed ARGs

According to the results of Enrichr database, in the aspect of biological processes, the genes were mostly enriched in apoptotic process, intrinsic apoptotic signaling pathway and intrinsic apoptotic signaling pathway in response to endoplasmic reticulum stress. In the aspect of cellular components, the genes were mostly enriched in mitochondrial outer membrane, mitochondrion, and autophagosome. In the aspect of molecular function, the genes were mostly enriched in protein heterodimerization activity, protein serine/threonine kinase inhibitor activity, and protein phosphatase binding. Besides, we also found the differentially expressed ARGs were notably associated with apoptosis, pathways in cancer, autophagy, Kaposi sarcoma-associated herpesvirus infection and measles in the KEGG pathway enrichment analysis (Additional file 1).

### Construction of a risk score formula in the train set

The relationships between the expression level of the 40 differentially expressed ARGs and overall survival (OS) were evaluated based on the data obtained from TCGA. Firstly, we integrated autophagy-related gene expression profiles and clinical follow-up information to screen out 516 EC samples. Then the entire set (516 samples) were randomly separated into the train set and the test set in a ratio of 6 to 4. Hence, the train set contained 312 samples and the test set contained 204 samples. Using the expression files of the train set, the univariate Cox regression, LASSO Cox regression and multivariate Cox regression analyses were performed (Additional file 2). A prognostic model using the expression of CDKN1B, DLC1, EIF4EBP1, ERBB2 and GRID1 was constructed (Table 2). The risk score of EC prognosis was quantified by the following formula: risk score = CDKN1B * (0.016941) + DLC1 * (− 0.33079) + EIF4EBP1 * (0.003577) + ERBB2 * (0.001271) + GRID1 * (0.733501). It was noticed that among these five ARGs, just DLC1 had a negative coefficient. Totally, the expressions of CDKN1B, EIF4EBP1, ERBB2 and GRID1 were negatively related to the OS of EC patients, while that of DLC1 was positively related to OS.

**Table 2:** The most prognosis-related five autophagy-related genes

Gene	Coef	HR	HR.95L	HR.95H	p-value
CDKN1B	0.016941	1.017085	0.994197	1.040501	0.144618
DLC1	− 0.33079	0.718355	0.542355	0.951468	0.02106
EIF4EBP1	0.003577	1.003584	1.000417	1.00676	0.026523
ERBB2	0.001271	1.001272	1.000166	1.002379	0.024188
GRID1	0.733501	2.082357	1.20684	3.59303	0.008402

### Relationships between the risk score and OS in the train set

**Fig. 2:**
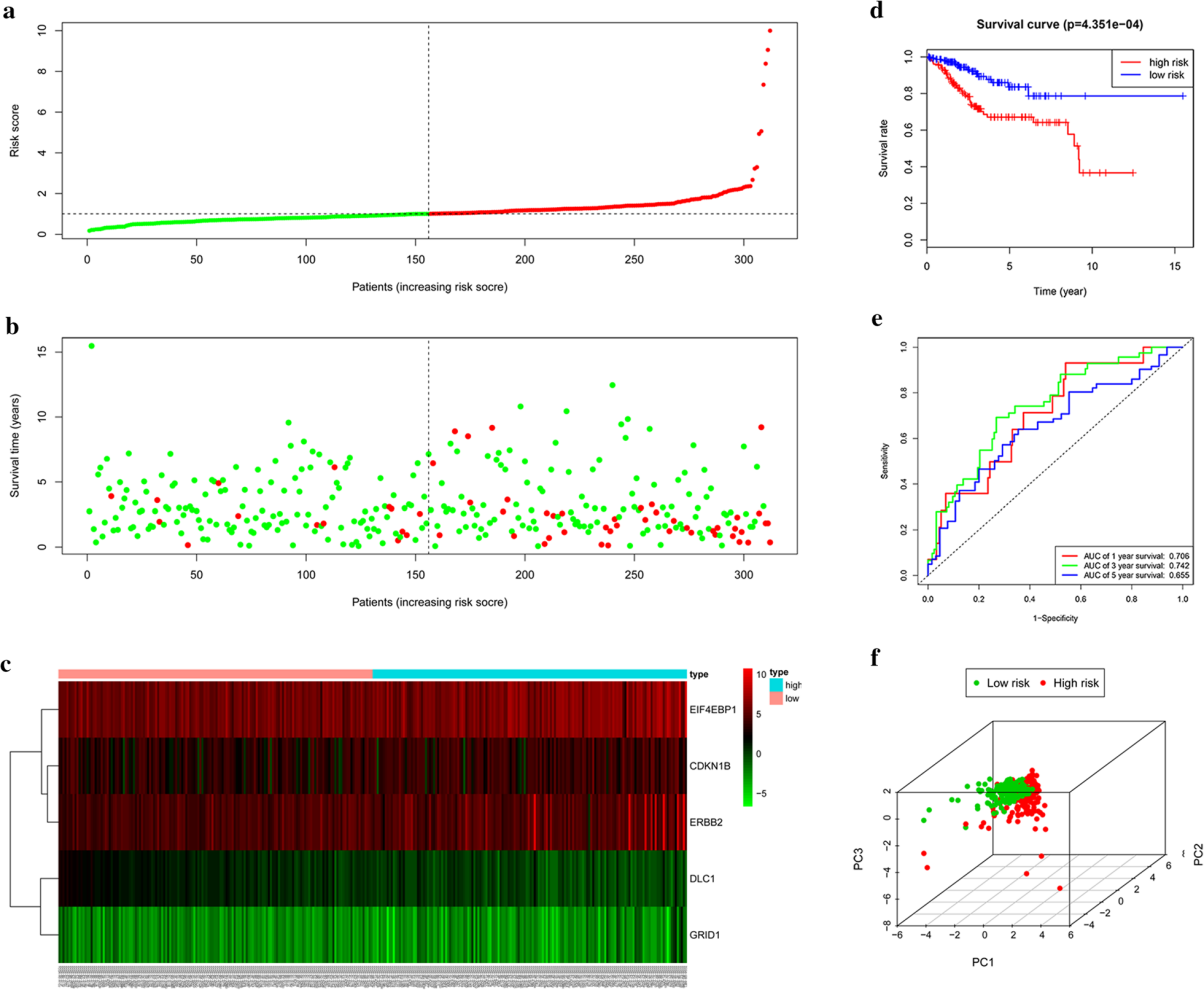
Prognostic analysis of the train set. **a** Rank of risk score and distribution of groups. **b** The overall survival of patients in different groups. **c** Expression heatmap. **d** Kaplan–Meier survival analysis. **e** Time-dependent ROC curve analysis for survival prediction by the risk score. **f** Principal component analysis

Based on these five ARGs, we could calculate the risk score for each patient. Survival analysis was performed and a dichotomous score was adopted. Patients in the train set were then stratified into high-risk (n = 156) and low-risk groups (n = 156) based on the median risk score. The risk score and survival status predicted by the prognostic model were displayed in Fig. 2a–c. The survival rate of the patients in the low-risk group was significantly higher than that in the high-risk group (*p *= 4.351e−04) (Fig. 2d). Time-dependent ROC analysis demonstrated that the prognostic accuracy of the risk score was 0.706 at 1 year, 0.742 at 3 years, and 0.655 at 5 years, indicative of its good performance in predicting EC prognosis (Fig. 2e). Principal component analysis displayed a different distribution pattern of high and low risk according to 5 autophagy-related gene expression based on the train set (Fig. 2f). Univariate and multivariate Cox regression analyses showed that the model can efficiently predict EC prognosis with ARG risk score as an independent indicator (Fig. 5a, b).

### Validation of the risk score in the test set

**Fig. 3:**
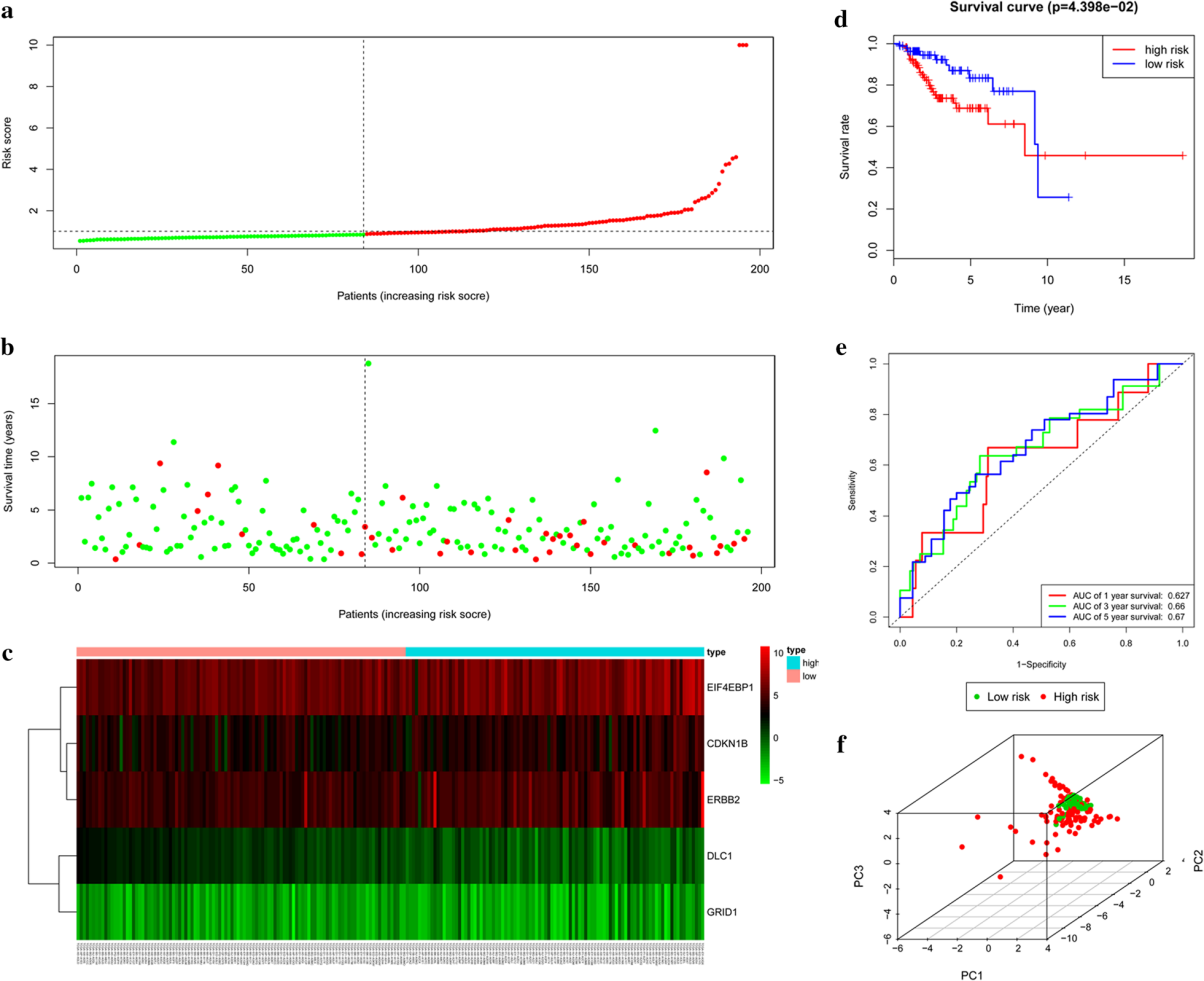
Prognostic analysis of the test set. **a** Rank of risk score and distribution of groups. **b** The overall survival of patients in different groups. **c** Expression heatmap. **d** Kaplan–Meier survival analysis. **e** Time-dependent ROC curve analysis for survival prediction by the risk score. **f** Principal component analysis

The test set was applied to validate the predictive power of the five ARGs. Based on the cut-offs in the train set, 204 samples in the test set were divided into low-risk (n = 107) and high-risk groups (n = 97) by using the same risk score formula. Figure 3a–c also showed the risk score and survival status. In consistent with the findings described above, low-risk patients had significantly longer overall survival than high-risk patients (p = 4.398e−02) (Fig. 3d). ROC curve analysis showed that the specificity and sensitivity were highest when the risk score was 0.627 at 1 year, 0.66 at 3 years, 0.67 at 5 years, according to the value of the area under the receiver operating characteristic curve (AUC) (Fig. 3e). Principal component analysis displayed a different distribution pattern of high and low risk according to 5 autophagy-related gene expression based on the test set (Fig. 3f). Likewise, the independency of the prognostic model was also confirmed by univariate and multivariate Cox regression analyses (Fig. 5c, d).

### Validation of the risk score in the entire set

**Fig. 4:**
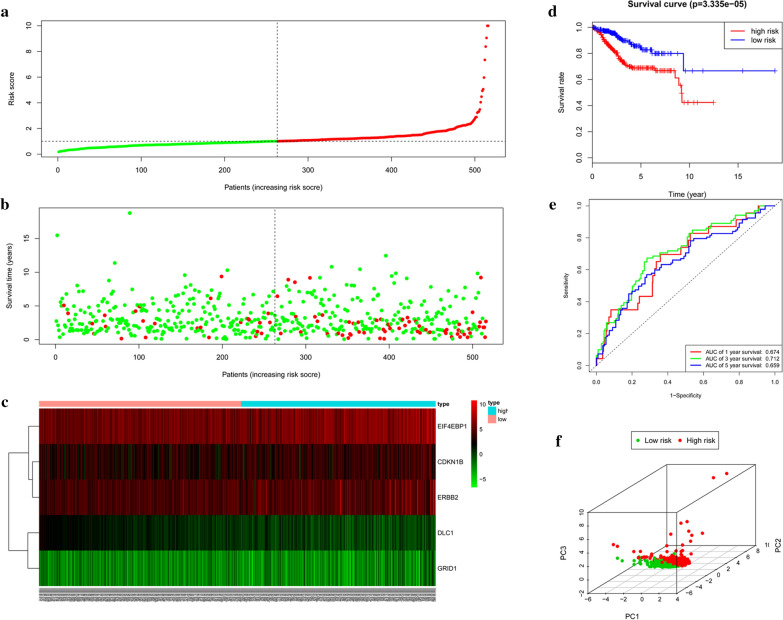
Prognostic analysis of the entire set. **a** Rank of risk score and distribution of groups. **b** The overall survival of patients in different groups. **c** Expression heatmap. **d** Kaplan–Meier survival analysis. **e** Time-dependent ROC curve analysis for survival prediction by the risk score. **f** Principal component analysis

**Fig. 5:**
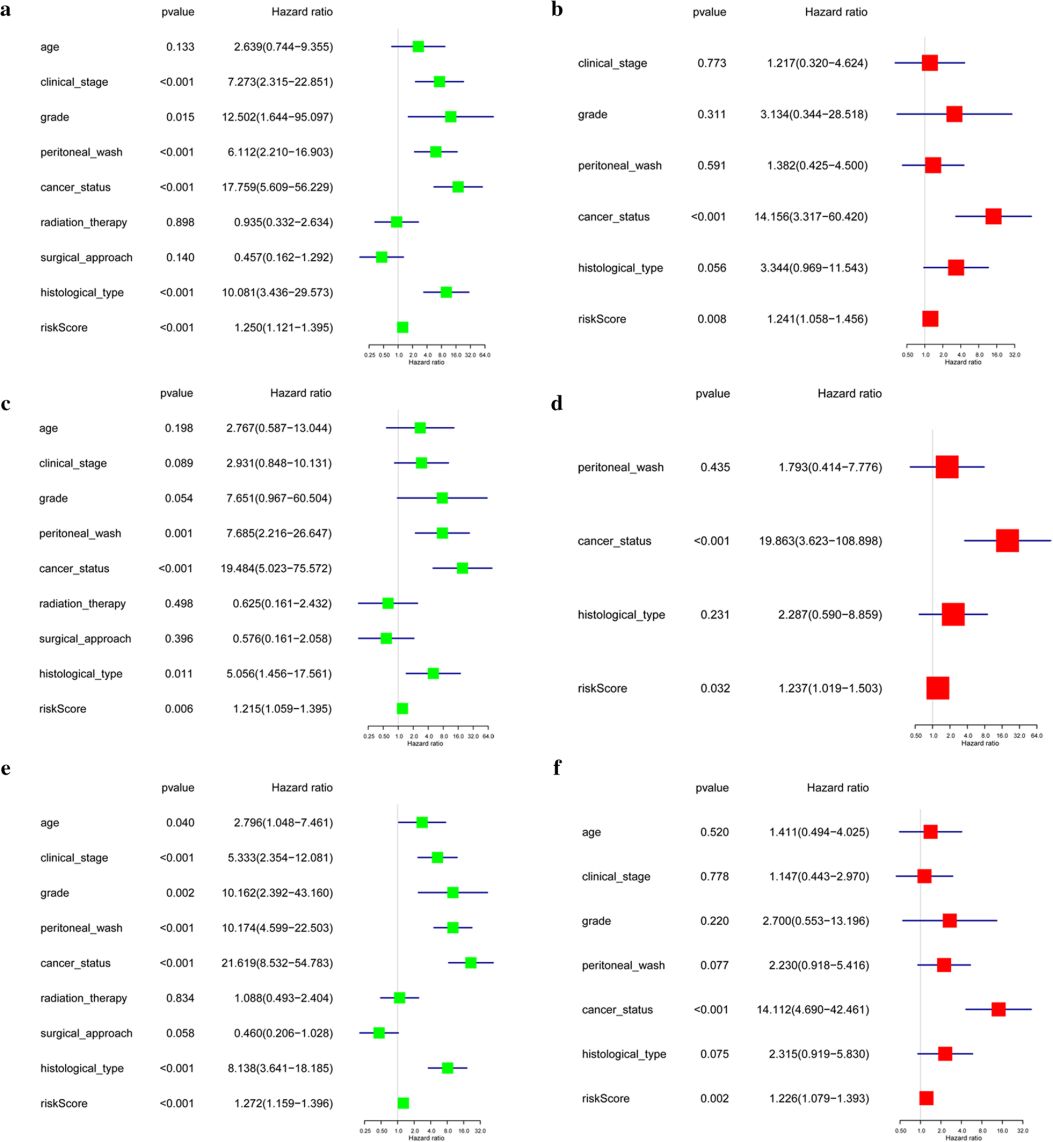
Univariate (**a**, **c**, **e**) and multivariate (**b**, **d**, **f**) regression analyses of the prognostic value for the train set (**a**, **b**), the test set (**c**, **d**) and the entire set (**e**, **f**) with clinicopathologic factors

We further validated our risk score in the entire set (516 samples). Based on the cut-offs in the train set, the samples were stratified into low-risk group (n = 263) and high-risk group (n = 253). Figure 4a–c also showed the risk score and survival status. KM plot indicated that patients in high-risk group and low-risk group showed significantly different outcomes (*p *= 3.335e−05) (Fig. 4d). The 1‐year, 3‐year and 5‐year prognostic accuracy of the prognostic model was 0.674, 0.712 and 0.659, respectively (Fig. 4e). Principal component analysis displayed a different distribution pattern of high and low risk according to 5 autophagy-related gene expression based on the entire set (Fig. 4f). The risk score and clinical factors were incorporated into univariate and multivariate Cox regression analyses. The results demonstrated that the ARG risk score in the model can serve as an independent prognostic indicator (Fig. 5e, f). These conclusions supported our speculation that autophagy affects the prognosis of EC.

### Relationships between the risk score and clinicopathologic factors

**Fig. 6:**
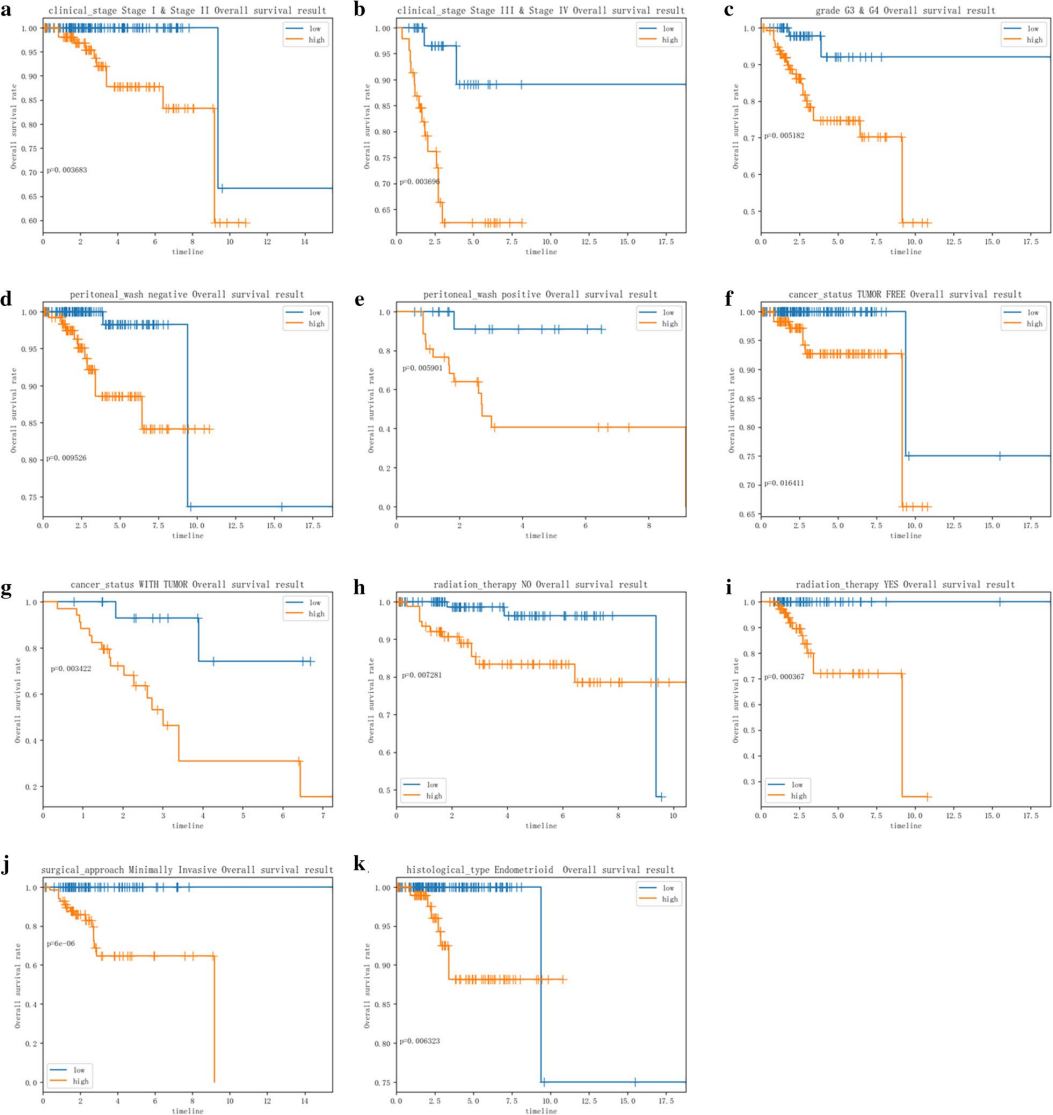
Stratified analyses of clinicopathological factors in EC: stage I, II & III, IV (**a**, **b**), grade G3&G4 (**c**), peritoneal wash (**d**, **e**), cancer status (**f**, **g**), radiation therapy (**h**, **i**), surgical approach (**j**), and histological type (**k**)

We finally built a complete prognostic model based on the entire set. This model showed a good performance in stratification in clinical stage I–II & III–IV, grade G3&G4, peritoneal wash, cancer status, radiation therapy, minimally invasive surgical approach and endometrioid histological type (Fig. 6). In parallel to the results above, high‐risk group in both subgroups was inclined to have worse OS. Meanwhile, relationships were analyzed between the ARGs-based risk score and clinicopathologic factors, including age, grade, clinical stage, peritoneal wash, radiation therapy, surgical approach, cancer status and histological type. Risk score was significantly higher in seniors, poorly differentiated cases, and mix or serous histological type cases (Additional file 3). We also explored the clinical significance of included genes (Table 3).

**Table 3:** Relations between the expression of the autophagy-related genes and the clinicopathological factors in endometrial cancer

Genes	Age (> 60/≤ 60)		Clinical stage (G1–G2/G3–G4)		Grade (I–II/III–IV)		Peritoneal wash (wash negative/positive)		Cancer status (with tumor/tumor free)		Radiation therapy (yes/no)		Surgical approach (minimally invasive/open surgery)		Histological type (endometrioid/mixed & serous)	
	t	p	t	p	t	p	t	p	t	p	t	p	t	p	t	p
CDKN1B	− 0.119	0.906	− 0.623	0.534	− 6.612	< 0.001	− 0.55	0.584	− 1.808	0.075	− 0.591	0.555	1.336	0.183	− 3.337	0.001
DLC1	4.998	< 0.001	− 0.024	0.981	3.947	< 0.001	1.019	0.313	3.575	< 0.001	1.234	0.218	0.555	0.580	4.223	< 0.001
EIF4EBP1	0.141	0.888	− 1.302	0.196	− 2.551	0.011	1.209	0.231	− 0.952	0.346	0.877	0.381	− 0.068	0.946	0.05	0.960
ERBB2	− 2.346	0.020	− 1.681	0.097	− 2.576	0.011	− 1.98	0.055	− 0.737	0.464	− 0.364	0.716	0.116	0.908	− 2.458	0.017
GRID1	2.203	0.029	− 1.352	0.179	1.092	0.276	0.425	0.672	0.281	0.779	0.202	0.840	− 0.668	0.505	− 0.681	0.497

t: t value of student’s test; p: p-value of student’s test

### Nomogram and its clinical utility

**Fig. 7:**
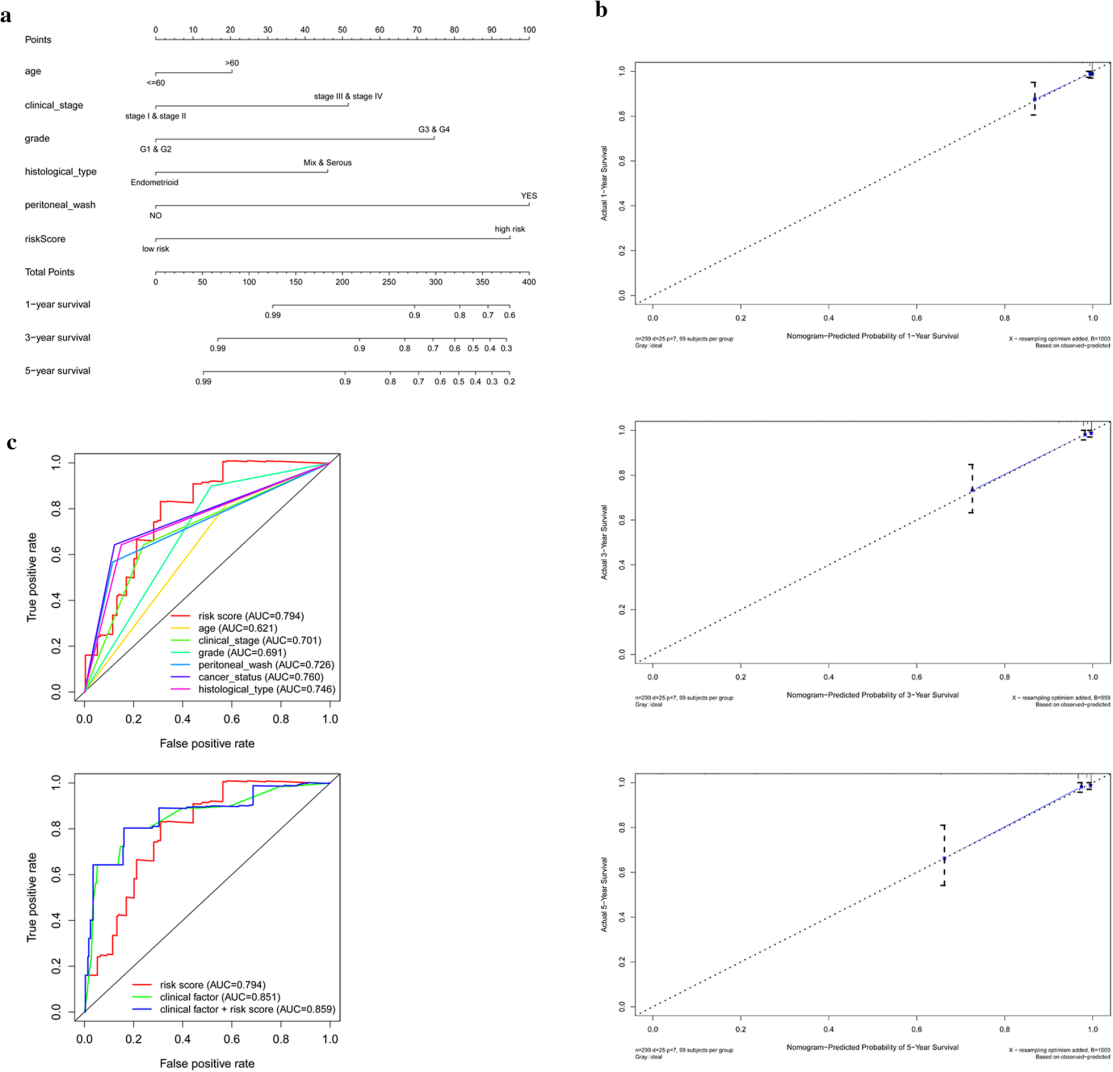
**a** The nomogram to predict 1-, 3- or 5-year OS in the entire set. **b** The calibration plots for predicting patient 1-, 3- or 5-year OS. **c** ROC curve analysis

To provide clinicians with a quantitative method to predict the overall survival of EC patients, a nomogram was constructed incorporating the risk score and clinical factors (Fig. 7a). The calibration curve of 1-year, 3-year and 5-year survival indicated that the nomogram was almost an ideal model (Fig. 7b). ROC curve analysis also showed that in this model, the risk score AUC was 0.794, and the clinical factors AUC was 0.851, both much significantly higher than that of patient age (AUC = 0.621), clinical stage (AUC = 0.701), grade (AUC = 0.691), peritoneal wash (AUC = 0.726), cancer status (AUC = 0.760) and histological type (AUC = 0.746). Interestingly, the ROC curve of risk score combined with clinical factors was much higher than that of each alone (AUC = 0.859) (Fig. 7c). These further validated the reliability of our previous prognostic model.

### External validation of the risk score

**Fig. 8:**
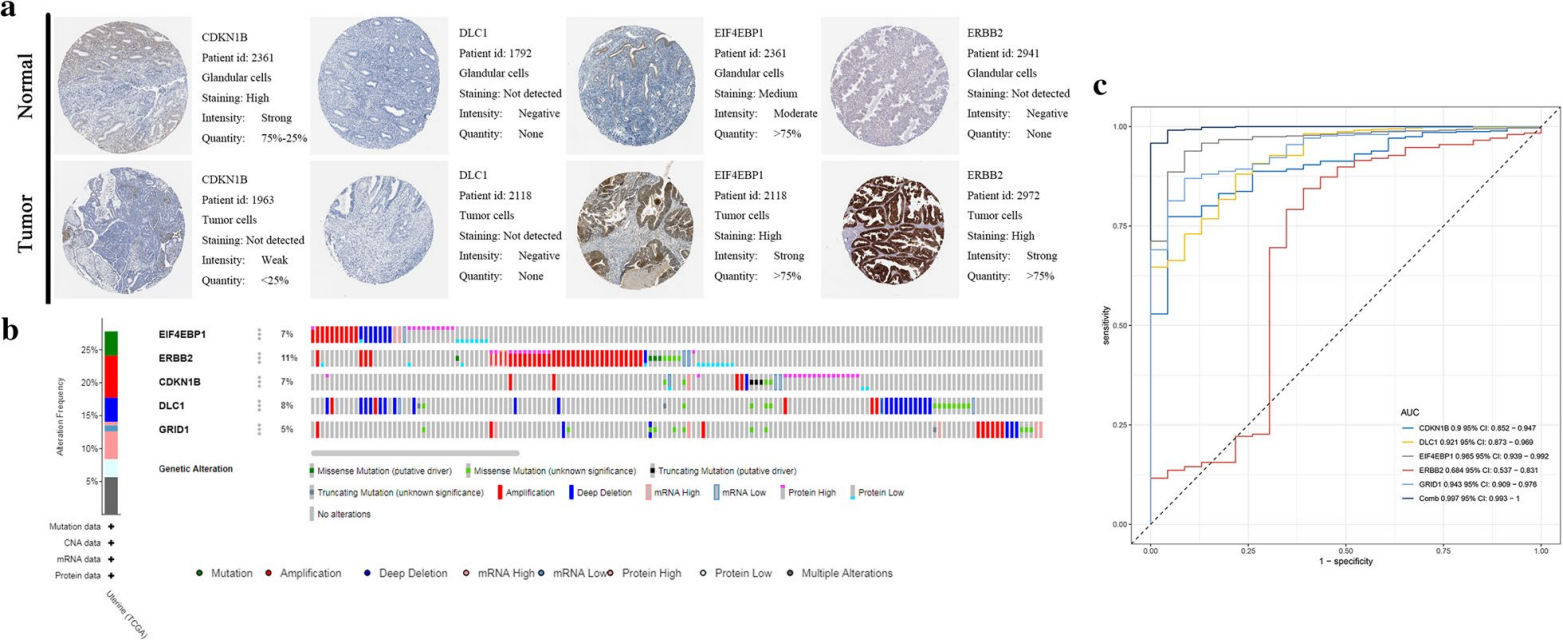
Expression and genetic alterations of the five predictive genes. **a** The representative protein expression of the five genes in EC and normal endometrium tissue. Data were from the Human Protein Atlas (http://www.proteinatlas.org) database. Data of GRID1 were not found in the database. **b** Genetic alterations of the five genes in TCGAUCEC. Data were from the cBioportal for Cancer Genomics (http://www.cbioportal.org/). **c** multi-AUC

The representative protein expression of CDKN1B, DLC1, EIF4EBP1 and ERBB2 was explored in the Human Protein Profiles (Fig. 8a). However, we failed to find GRID1 on the website. ERBB2 possessed the most genetic alterations (11%), amplification being the commonest (Fig. 8b). Moreover, ROC curve analysis found that the key genes had strong capacity to distinguish tumor from normal tissues, and the combined use of these ARGs was more effective (AUC = 0.997) (Fig. 8c).

### Gene set enrichment analysis

We performed GSEA to identify ARGs related pathways. The most enriched pathways in high-risk groups included “Cell cycle”, “Citrate cycle tca cycle”, “DNA replication”, “Homologous recombination”, “Mismatch repair” and “Oxidative phosphorylation”, which implied that the five ARGs are related to EC progress (Additional file 4).

### Somatic mutation analysis

**Fig. 9:**
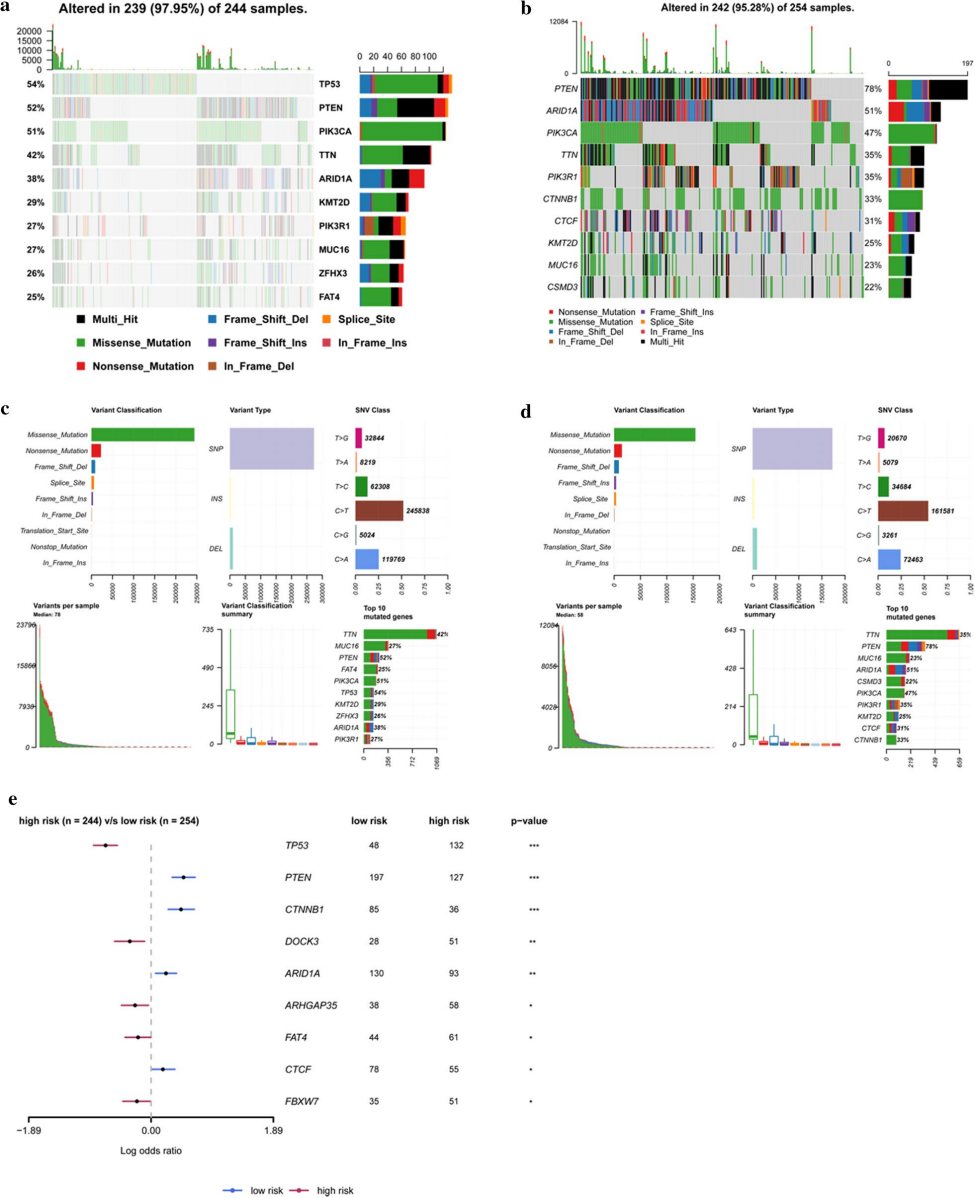
Somatic mutation analysis. **a** The top ten mutated genes in high-risk group. **b** The top ten mutated genes in low-risk group. **c**, **d** Cohort summary plot displaying distribution of variants according to variant classification, type, and SNV class. Bottompart (from left to right) indicates mutation load for each sample, variant classification type A stacked barplot shows top ten mutated genes in high-risk and low-risk group. **e** mutated genes differentially between the high- and low-risk groups

The top ten mutated genes in different risk groups were displayed in the Fig. 9. The order of somatic mutations in the high-risk group was as follows: TP53 > PTEN > PIK3CA > TTN > ARID1A > KMT2D > PIK3R1 > MUC16 > ZFHX3 > FAT4 (Fig. 9a). In the low-risk group, the order was: PTEN > ARID1A > PIK3CA > TTN > PIK3R1 > CTNNB1 > CTCF > KMT2D > MUC16 > CSMD3 (Fig. 9b). Besides, we found that the patients in the high-risk group showed significant mutation signatures, compared with those in the low-risk group (Fig. 9c, d). It was noticed that TP53, PTEN, CTNNB1, DOCK3, ARID1A, ARHGAP35, FAT4, CTCF and FBXW7 were mutated differentially between the high- and low-risk groups (p < 0.05) (Fig. 9e).

### Expression levels of ARGs in clinical tissue samples

**Fig. 10:**
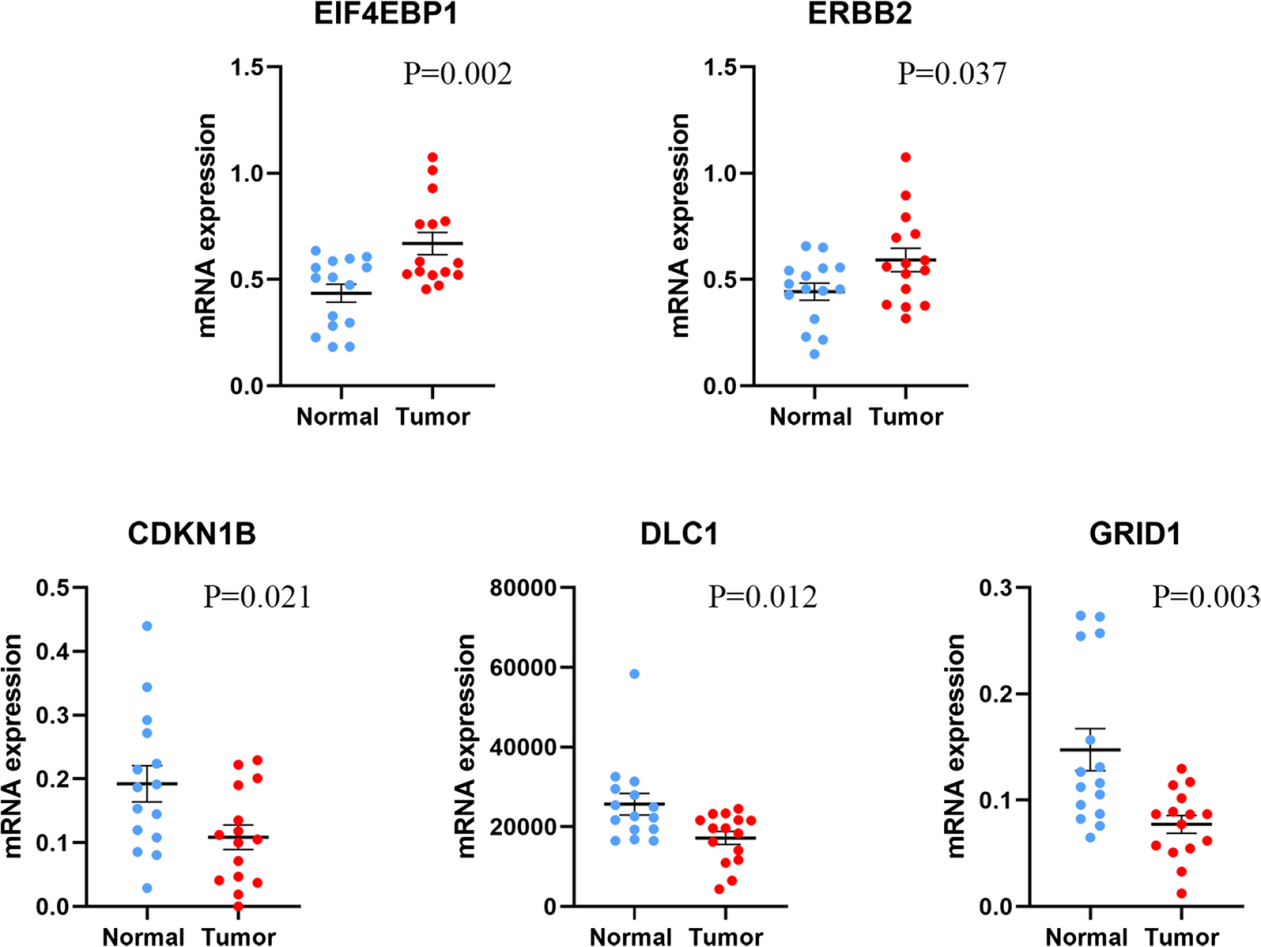
Expression levels of the five ARGs mRNA in clinical tissue samples

Finally, the qRT-PCR results from our own specimens verified that the expression of EIF4EBP1 and ERBB2 mRNA were significantly higher in EC tissues than that in normal endometrial tissues, while the expression of CDKN1B, DLC1 and GRID1 mRNA were lower. The p values were 0.002, 0.037, 0.021, 0.012 and 0.003 respectively (Fig. 10).

## Discussion

Endometrial cancer is a major gynecological malignancy worldwide. Prognosis-related high risk factors include disease stage, tumor size, grade, histological type, myometrial invasion and lymph node metastasis [14]. Nevertheless, these risk factors could only be determined after extensive surgery. Consequently, preoperative prognostic factors are needed, especially for those who are not suitable for surgery or wish to preserve their fertility function. Similar to other tumors, the occurrence, development and metastasis of EC also involve complex molecular mechanism [15]. Thus, reliable molecular biomarkers predicting the prognosis of EC are significant for selecting patients who might be sensitive to additional targeted therapy [16]. Autophagy is a highly conserved catabolic and energy-generating process that maintains cell homeostasis through degrading damaged organelles or intracellular components [17]. It was reported that autophagy plays complicated and momentous roles in EC genesis and progression. In type I endometrial cancer, as autophagy is repressed by activated PI3K/AKT/mTOR signaling pathway, the proliferation of tumor cells becomes facilitated, indicating that autophagy inhibits the development of tumors. Liu et al. showed that activation of the PI3K/AKT pathway could result in activation of mTOR and the proliferation of endometrial cells by inhibiting autophagy [18]. Currently, autophagy is considered as a “double-edged sword”, since it not only inhibits tumor progression by depriving cells of nutrients and energy, but also promotes tumor progression by conferring cells with nutrients and energy [19, 20]. Therefore, autophagy-related research may open a new window into EC pathogenesis and prognosis. Several autophagy-related prognostic indicators had already been proposed. For example, An et al. developed an eight-gene autophagy-related signature, which could serve as an independent prognostic indicator for serous ovarian cancer [21]. Wang et al. identified three prognostic ARGs (JUN, MYC, and ITGA3) and validated a clinical autophagy-based index in predicting overall survival of bladder cancer [22]. However, the prognostic significance of autophagy genes in endometrial cancer has not been reported.

In the present study, we first screened differentially expressed ARGs in EC. Subsequently, several gene functional enrichment analyses were performed to define their biological process, molecular function and signaling pathways. Via univariate and multivariate survival analysis, we identified that CDKN1B, DLC1, EIF4EBP1, ERBB2 and GRID1 were significantly related to EC patients’ overall survival. A prognostic model was constructed based on them, all of which showed potential of being therapeutic targets. CDKN1B encodes p27Kip1 protein, a cyclin-dependent kinase inhibitor that binds to and prevents the activation of a broad range of cyclin CDK complexes, thereby controlling the cell cycle progression at G1 [23]. Recent studies have demonstrated that CDKN1B can be genetically inactivated, particularly in luminal breast cancer, prostate cancer, and small intestine neuroendocrine neoplasms [24]. DLC1 gene encodes GTPase-activating protein (GAP), a member of the Rho-GAP family of proteins, and plays a role in the regulation of small GTP-binding proteins [25]. GAP proteins are involved in signaling pathways that regulate cellular processes in cytoskeletal changes, functioning as a tumor suppressor in a variety of common cancers, such as lung cancer, liver cancer, colorectal cancer, breast cancer and prostate cancer [26]. This role is consistent with our findings. EIF4EBP1 encodes one translation repressor protein, which directly interacts with eukaryotic translation initiation factor 4E (eIF4E), to inhibit complex assembly and repress translation. This protein is phosphorylated in response to various signals, resulting in its dissociation from eIF4E and activation of mRNA translation [27, 28]. High level of EIF4EBP1 phosphorylation is associated with malignant progression and adverse prognosis in several malignancies, such as breast, ovary and prostate cancers [29]. ERBB2 encodes an epidermal growth factor (EGF) receptor and binds tightly to other ligand-bound EGF receptors to form a heterodimer, stabilizing ligand binding and enhancing kinase-mediated activation of downstream signaling pathways, such as phosphatidylinositol-3 kinase and mitogen-activated protein kinase. Amplification and/or overexpression of this gene have been reported in lots of cancers, including ovarian and breast cancers [30]. ERBB2 overexpression shows association with EC prognosis [31, 32]. GRID1 is a member of glutamate receptors, which were thought to exclusively play a role in the central nervous system (CNS). However, it was recently suggested that glutamate receptor subunits are also involved outside the CNS in malignant processes, such as pancreatic cancer, breast cancer and ovarian cancer [33, 34]. From a clinical perspective elevated expression in high-risk endometrial cancers makes the gene, which is reported in the present study for the first time attractive as a potential tumor marker.

Our gene enrichment analysis also confirmed their involvement in cancer-related biological processes. Besides, higher rates of TP53, ZFHX3 and FAT4 mutation were found in high-risk patients than low-risk patients. TP53 is a critical tumor suppressor that regulates cell cycle progression, apoptosis, cell senescence and many others. Its mutation evokes diverse physiological effects, including numerous cancers [35]. Therefore, TP53 mutation can be used to guide the design of new EC treatments. Our GSEA analysis suggested that high risk score tended to be accompanied by a number of up-regulated networks, including tumorigenesis and tumor progression associated pathways. For example, instability is a hallmark of cancer, most likely caused by abnormal DNA replication [36]. Homologous recombination enables the cell to obtain and replicate intact DNA sequence information, especially to repair DNA damage. Patients with homologous recombination deficiencies were more responsive to treatments, such as platinum-based chemotherapy and poly (ADP-ribose) polymerase (PARP) inhibitors [37, 38].

Using TCGA, we screened out autophagy-related genes and used them to build up a prognostic risk score model. The model displayed powerful ability to predict the survival in patients stratified according to ages, grades, clinical stages and histological types. Besides, the model was validated using the test set and the entire set. Thus, it might be converted into clinical application.

However, there are some limitations in this study. First, the underlying molecular mechanism of the key ARGs in the EC pathogenesis were undefined. Second, other potential prognostic variables associated with EC, such as tumor size, myometrial invasion and lymph node metastasis, should be investigated. Third, prospective studies are required to further confirm the clinical utility and the biological function of this signature.

## Conclusions

In conclusion, based on five autophagy-related genes (CDKN1B, DLC1, EIF4EBP1, ERBB2 and GRID1), our model can independently predict the OS of EC patients by combining molecular signature and clinical characteristics.

## Supplementary information

**Additional file 1.** GO and KEGG analysis of differentially expressed ARGs. GO, gene ontology; KEGG, Kyoto Encyclopedia of Genes and Genomes.

**Additional file 2.** Identification of prognosis related ARGs using LASSO regression model. (A) LASSO coefficient profiles of the ARGs associated with the overall survival of endometrial cancer. (B) Plots of the cross-validation error rates. Each dot represents a lambda value along with error bars to give a confidence interval for the cross-validated error rate.

**Additional file 3.** Relationships between the risk score and (A) age; (B) grade; (C) histological type.

**Additional file 4.** GSEA showed significant enrichment hallmarks in high-risk versus low-risk group. (A) Cell cycle; (B) Citrate cycle tca cycle; (C) DNA replication; (D) Homologous recombination; (E) Mismatch repair; (F) Oxidative phosphorylation.

## Data Availability

The data sets used and/or analyzed during the current study are publicly available data from The Cancer Genome Atlas (TCGA). The figures and materials supporting the conclusions of this article are included within the article and its additional files).

## Abbreviations

ARGs: Autophagy-related genes
AUC: Area under the curve
CNS: Central nervous system
EC: Endometrial cancer
GO: Gene ontology
GSEA: Gene Set Enrichment Analysis
KEGG: Kyoto Encyclopedia of Genes and Genomes
MAF: Mutation annotation format
MSigDB: Molecular signature database
OS: Overall survival
qRT-PCR: Quantitative real-time PCR
RNA-seq: RNA sequencing
ROC: Receiver operating characteristic
SLS: Stone-like structure
TCGA: The Cancer Genome Atlas
