# Supplementary figures and images for: Prognostic value of an autophagy-related gene expression signature for endometrial cancer patients
Source: Cancer Cell Int. 2020 Jul 13;20:306. doi: 10.1186/s12935-020-01413-6 (PMC7359499; doi:10.1186/s12935-020-01413-6)

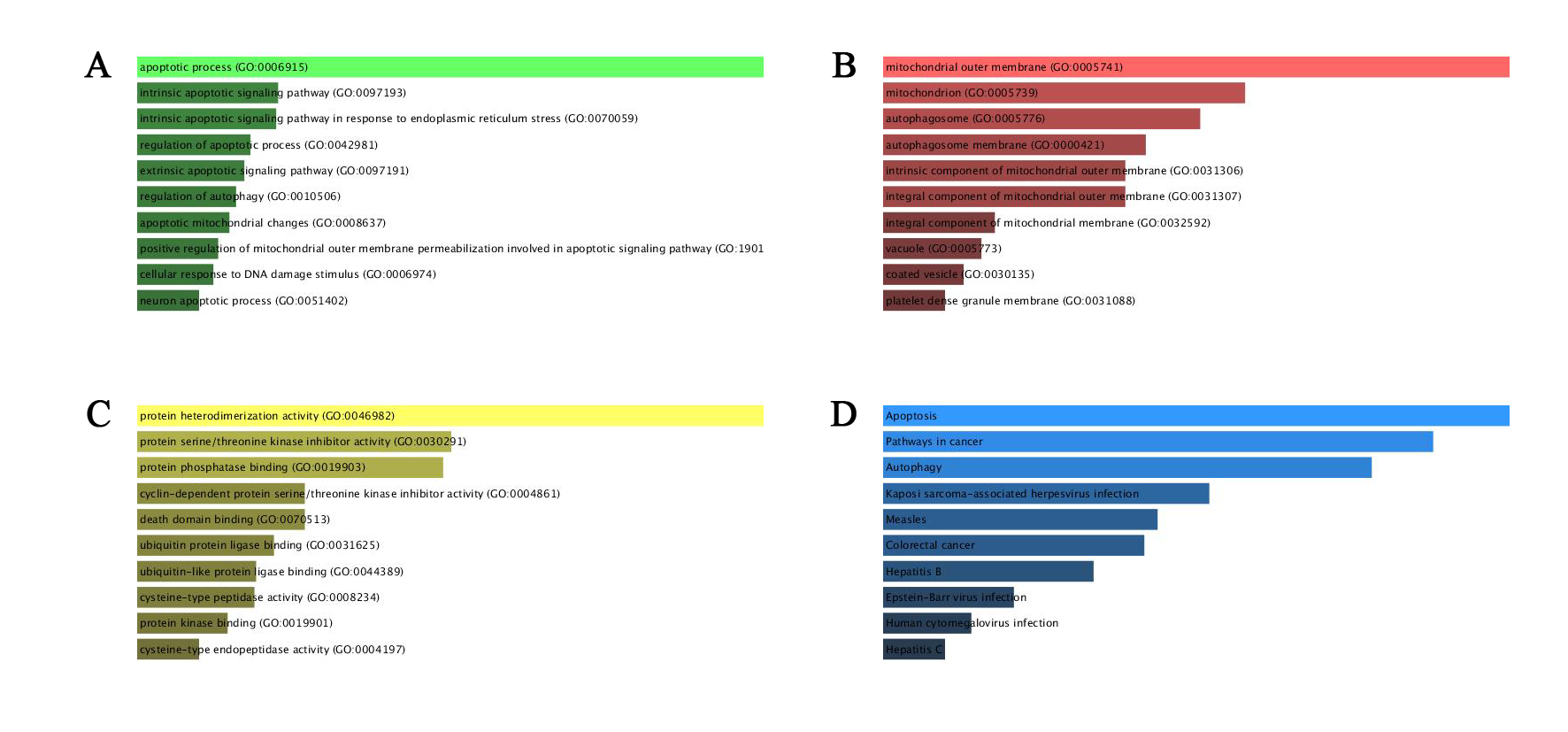

Supplement: Supplementary file 1 — Additional file 1. GO and KEGG analysis of differentially expressed ARGs. GO, gene ontology; KEGG, Kyoto Encyclopedia of Genes and Genomes. [file 12935_2020_1413_MOESM1_ESM.tif]

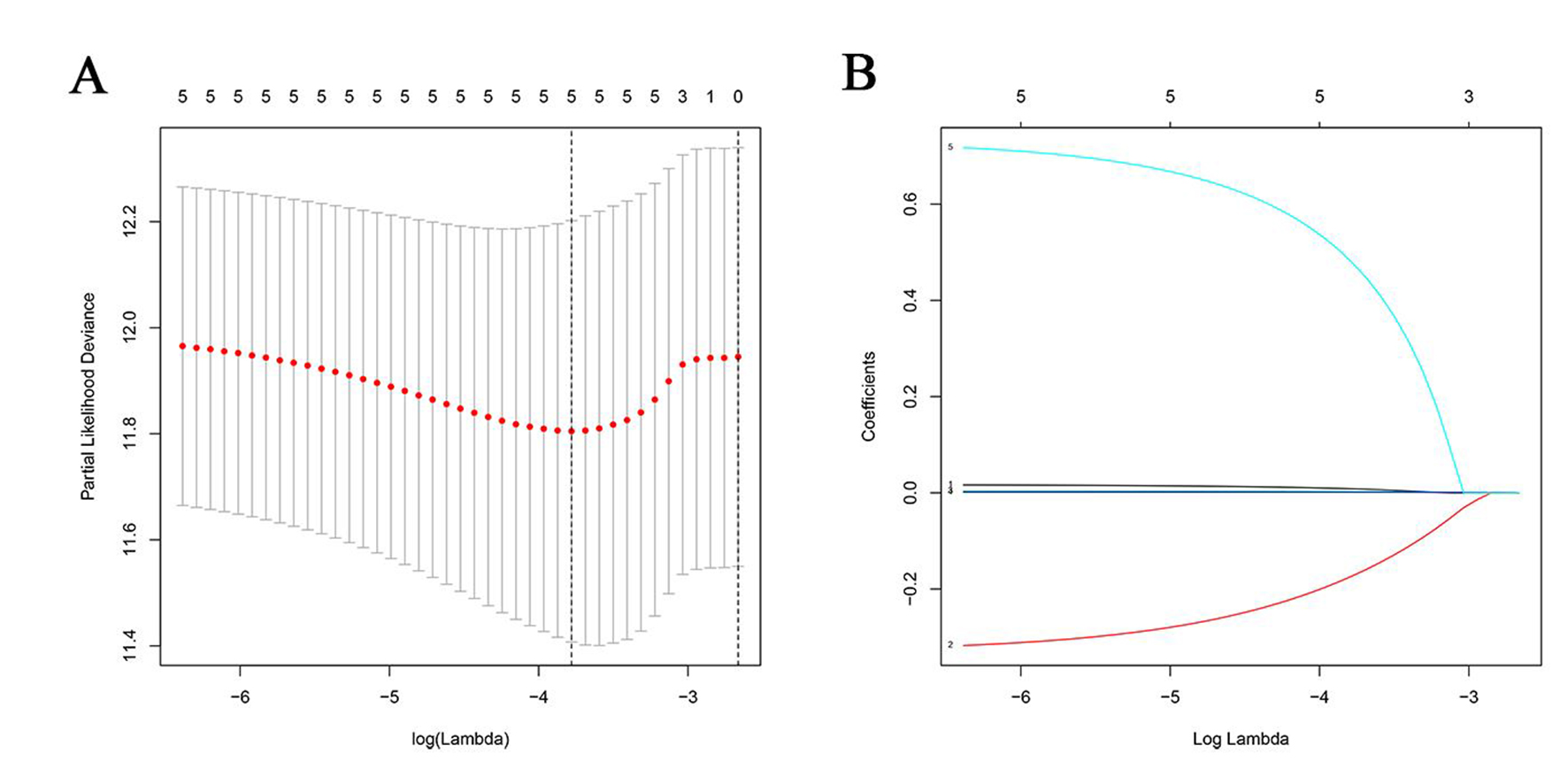

Supplement: Supplementary file 2 — Additional file 2. Identification of prognosis related ARGs using LASSO regression model. (A) LASSO coefficient profiles of the ARGs associated with the overall survival of endometrial cancer. (B) Plots of the cross-validation error rates. Each dot represents a lambda value along with error bars to give a confidence interval for the cross-validated error rate. [file 12935_2020_1413_MOESM2_ESM.jpg]

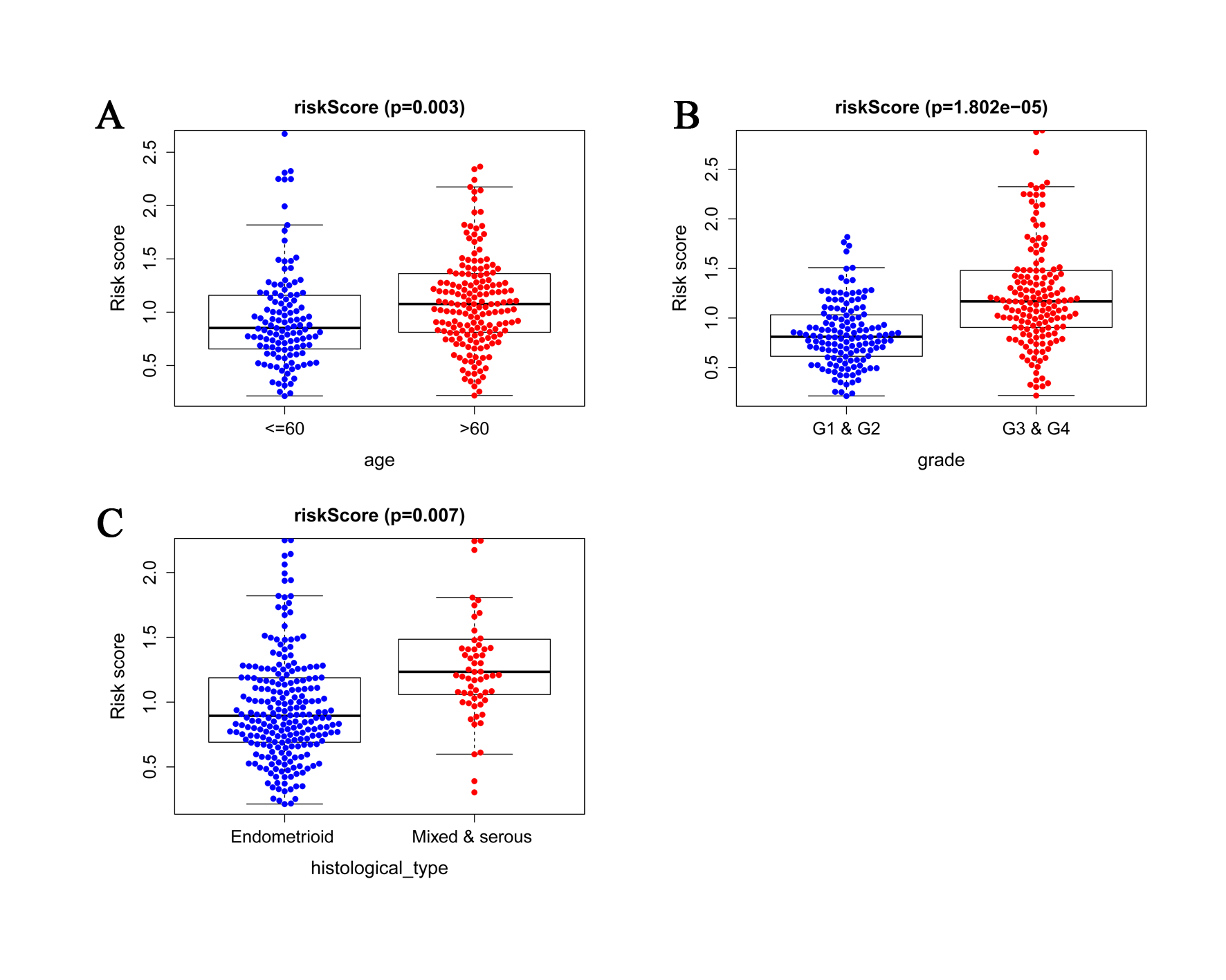

Supplement: Supplementary file 3 — Additional file 3. Relationships between the risk score and (A) age; (B) grade; (C) histological type. [file 12935_2020_1413_MOESM3_ESM.tif]

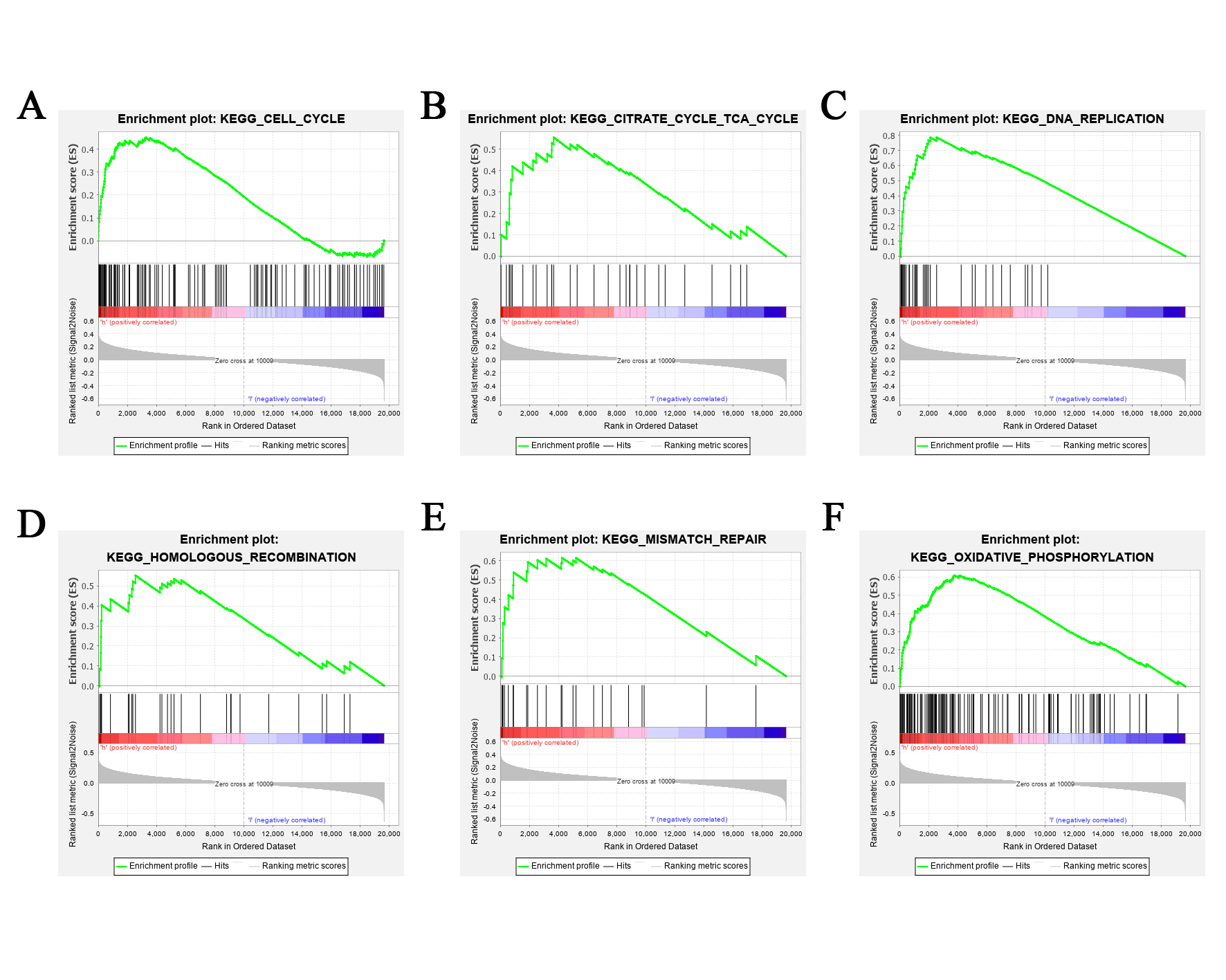

Supplement: Supplementary file 4 — Additional file 4. GSEA showed significant enrichment hallmarks in high-risk versus low-risk group. (A) Cell cycle; (B) Citrate cycle tca cycle; (C) DNA replication; (D) Homologous recombination; (E) Mismatch repair; (F) Oxidative phosphorylation. [file 12935_2020_1413_MOESM4_ESM.tif]
